# Chemoprevention of Lung Cancer: Prospects and Disappointments in Human Clinical Trials

**DOI:** 10.3390/cancers5010131

**Published:** 2013-01-24

**Authors:** Alissa K. Greenberg, Jun-Chieh Tsay, Kam-Meng Tchou-Wong, Anna Jorgensen, William N. Rom

**Affiliations:** Division of Pulmonary, Critical Care, and Sleep Medicine, Departments of Medicine and Environmental Medicine, New York University School of Medicine, New York, NY 10016, USA

**Keywords:** lung cancer, chemoprevention, antioxidants, anti-inflammatories, carcinogenesis, biomarkers

## Abstract

Decreasing the risk of lung cancer, or preventing its development in high-risk individuals, would have a huge impact on public health. The most effective means to decrease lung cancer incidence is to eliminate exposure to carcinogens. However, with recent advances in the understanding of pulmonary carcinogenesis and the identification of intermediate biomarkers, the prospects for the field of chemoprevention research have improved dramatically. Here we review the most recent research in lung cancer chemoprevention—focusing on those agents that have been investigated in human clinical trials. These agents fall into three major categories. First, oxidative stress plays an important role in pulmonary carcinogenesis; and therefore, antioxidants (including vitamins, selenium, green tea extracts, and isothiocyanates) may be particularly effective in preventing the development of lung cancer. Second, inflammation is increasingly accepted as a crucial factor in carcinogenesis, and many investigators have focused on anti-inflammatory agents, such as glucocorticoids, NSAIDs, statins, and PPARγ agonists. Finally, the PI3K/AKT/mTOR pathway is recognized to play a central role in tobacco-induced carcinogenesis, and inhibitors of this pathway, including myoinositol and metformin, are promising agents for lung cancer prevention. Successful chemoprevention will likely require targeting of multiple pathways to carcinogenesis—both to minimize toxicity and maximize efficacy.

## 1. Introduction

Lung cancer is the most common cause of cancer death world-wide. This remains true, despite advances in the understanding of the molecular carcinogenesis and the development of new targeted therapies. In an attempt to decrease this high mortality rate, much attention has been devoted to developing methods for early detection of the disease, when it is at a more treatable stage. The National Lung Screening Trial (NLST) recently demonstrated that CT screening can decrease lung cancer mortality in high-risk smokers by 20% [1]. Many ongoing studies are looking at non-invasive biomarkers for early detection that could be combined with CT screening to decrease mortality even further. These methods may result in improved lung cancer survival; however, no screening program can succeed at detecting early stage cancer with 100% sensitivity and specificity. In addition, the five-year mortality rate for early stage (I and II) lung cancer is still over 50% [2], and the treatment for early stage lung cancer is surgical resection—resulting in significant morbidity. Decreasing the risk of lung cancer—or preventing its development in high-risk individuals—would be an even loftier goal.

## 2. Risk Reduction

The most obvious method to prevent lung cancer development is to eliminate the contributing risk factors for lung cancer. Smoking plays a role in over 90% of lung cancers. While exposures to other carcinogens (e.g., asbestos, radon, industrial chemicals, *etc*.), genetic predisposition, presence of COPD or emphysema, and some pulmonary infections also increase risk, lung cancer was exceedingly rare before the advent of cigarettes.

Archeological evidence indicates that the tobacco plant was first cultivated by the Maya in South America, perhaps as early as 5,000 BCE [3]. Christopher Columbus’ crew learned to smoke tobacco from the Arawak Indians and carried the habit back to Europe. Tobacco use then gradually spread throughout the world. However, until the manufacturing of cigarettes became widespread in the early 20th century, tobacco smoking was uncommon. In fact, smoking was illegal in many US states until the 1920s. The first report of lung cancer in medical literature appeared in the 18th century; and by 1898, only 140 cases had been reported [4]. In 1912, physicians were beginning to notice an increase in incidence, but it was still considered “among the rarest forms” of malignancy, and only 374 cases had been reported [5]. James Alexander Miller, the first Director of the Bellevue Chest Service in New York City, reviewed primary carcinoma of the lung in 1930 [6]. He presented 32 cases from Bellevue Hospital, and was among the first to link the disease to urban dust and bronchial irritation. Through the remainder of the 20th century the incidence of lung cancer skyrocketed—following the trend in smoking habits, with a latency of 25 years.

Smoking cessation is the only intervention that has yet been shown to prevent lung cancer. In the Lung Health Study smokers were randomly assigned to smoking cessation programs or no intervention [7]. The successful quitters had a 55% reduction in lung cancer incidence compared to the control group. However, despite progress in tobacco control, and smoking cessation campaigns, almost 20% of the adult population in the US continues to smoke. In some areas of the world the rates are much higher, and many more people are former smokers, or have had exposure to second-hand smoke [8]. The incidence of lung cancer in never-smokers has also increased, and is now up to 20 per 100,000 person-years [9]. This trend is less easily explained, but likely reflects both second-hand smoke exposure and an increase in other environmental carcinogens.

## 3. Chemoprevention

Chemoprevention is the use of dietary or pharmaceutical agents to prevent the development or progression of a disease. Only individuals at high risk for a condition should be treated, and treatment should commence before the disease develops, or when it is in its earliest stages. Since the risk-factors for lung cancer are well established, lung carcinogenesis may take 20–30 years, and many of the molecular events in lung carcinogenesis and some precursor lesions have been identified, this is an ideal disease for chemoprevention.

There are several types of chemoprevention for lung cancer. Primary chemoprevention is used to try to prevent the development of the disease in a high-risk population. Secondary chemoprevention aims to prevent the progression of precursor lesions to malignancy. Tertiary chemoprevention is directed at preventing recurrences or second cancers in individuals with prior aero-digestive cancers. Over 11% of smokers eventually develop lung cancer, and this would be the population to target for primary chemoprevention [10]. Identifying a population with pulmonary preneoplasia for secondary chemoprevention is more difficult. CT scans can identify small nodules, particularly sub-solid nodules that may represent preneoplasia. Sputum, buccal scrapings, nasal swabs or bronchoscopy can be used to identify genetic, epigenetic or other changes associated with the presence of preneoplasia or the development of malignancy; though these techniques are not yet validated. Patients with prior aero-digestive malignancies, the target group for tertiary chemoprevention, have a risk of developing a second primary lung cancer after resection of as high as 2–3% per year [11]. Depending on whether an agent is to be used for primary, secondary, or tertiary prevention, the acceptable level of toxicity may differ.

Since invasive cancer takes many years to develop, the identification of surrogate intermediate biomarkers to indicate success or failure of a potential agent are necessary for chemoprevention studies. Currently there is no gold-standard biomarker. The most commonly used markers are histologic changes consistent with preneoplasia based on a multi-step progression from hyperplasia, to metaplasia, to dysplasia, to carcinoma *in situ*, to squamous-cell lung cancer; and from atypical adenomatous hyperplasia to adenocarcinoma [12]. An accumulation of various genetic abnormalities correlates with the increasing morphologic changes, in both the squamous-cell carcinoma and the adenocarcinoma sequence. These genetic abnormalities associated with preneoplasia progression represent another class of biomarkers that can be used in chemoprevention studies. The sequence of events in the development of small-cell lung cancer is much less clearly defined, and chemoprevention studies for small cell lung cancer are more difficult to design. The development of validated markers of early pulmonary carcinogenesis is essential for successful clinical trials of chemopreventive agents. 

## 4. Agents for Lung Cancer Chemoprevention

No chemopreventive agent has been validated for lung cancer, though there are several promising areas of investigation. Some of the agents that have been studied in human clinical trials, especially those that have been investigated most recently, are reviewed here.

### 4.1. Antioxidants

Oxidative stress plays an important role in pulmonary carcinogenesis [13]. Oxidants or reactive oxygen species (ROS) are agents that form reactive oxygen metabolites, including hydroxyl radicals, superoxide anions, singlet oxygen and hydrogen peroxide. Antioxidants are substances that act to detoxify these species. Oxidative stress occurs if the balance of oxidants and antioxidants is disrupted. The major endogenous sources of oxidants are aerobic metabolism and inflammation. Exogenous sources include radiation, UVB light, atmospheric oxidants, tobacco smoke, manufactured fibers and various metals. Oxidants can induce carcinogenesis through several mechanisms, including direct DNA damage leading to structural damage or genetic mutation, altered gene expression due to a variety of epigenetic factors, activation or inhibition of signal transduction pathways, and inhibition of the apoptotic pathway. The respiratory tract is unique in the variety and extent of both endogenous and exogenous oxidants to which it is exposed. Therefore oxidative stress may play a particularly significant role in pulmonary carcinogenesis. As a result, it has been thought that antioxidants may be particularly effective in preventing the development of lung cancer.

Some of the antioxidants investigated as chemopreventive agents include vitamins A and E, selenium, *N*-acetylcysteine, and inducers of glutathione-S-transferase. Vitamin A was one of the first agents to be formally studied as a cancer chemopreventive agent. In 1925, Wolbach reported that rats developed epithelial neoplasia after vitamin A deprivation, and that this was reversed with supplementation [14]. Epidemiological data has suggested that diets high in vitamin A (retinols and carotenoids), vitamin E (tocopherols) and vitamin C reduce the risk of lung cancer [15,16,17]. When analogues of vitamin A (retinoids) that had lower toxicity were developed, studies with high dose supplementation could be pursued.

However, multiple trials in primary and secondary prevention—looking at combinations of β-carotene, retinol, alpha tocopherol, retinyl palmitate, and 13-*cis*-retinoic acid—did not show any protective effect [18,19,20,21,22,23]. Two studies in particular published in the *New England Journal of Medicine* in 1996 debunked the idea that β-carotene supplementation could be used to prevent lung cancer. In the Physicians' Health Study, more than 22,000 U.S. male doctors were treated with 50 mg of β-carotene or placebo every other day, for an average of 12 years [24]. There was no difference in the incidence of lung cancer mortality related to beta carotene supplementation. In the Beta-Carotene and Retinol Efficacy Trial (CARET), more than 18,000 persons at elevated risk for lung cancer because of exposure to asbestos or cigarette smoking were treated daily with beta carotene (30 mg) and retinyl palmitate (25,000 IU), or with placebo, for an average of four years [25]. The trial was ended early when the researchers identified an increased risk of death from lung cancer in the group receiving the supplements. A meta-analysis of the large beta carotene trials confirmed an increased risk of cancer in current smokers who received high-dose supplementation [26]. Phase III chemoprevention trials that looked at combinations of isotretinoin, vitamin A, *N*-acetylcysteine and/or selenium in patients with prior cancer showed no reduction in cancer recurrence [19,27,28]. A Cochrane Review of nine randomized controlled trials of vitamin and mineral supplementation for the prevention of lung cancer found no benefit for any of the combinations of high-dose supplements [29]. Most recently, investigators on the Physicians’ Health Study reported a slight decrease in total cancer diagnoses among physicians randomized to a multivitamin supplement, but there was no significant difference in lung cancer incidence [30]. Studies of several other antioxidants have also been negative. For example: anethole dithiolethione—an organosulfur compound that increases glutathione-S-transferase and additional phase II enzymes—did not reverse bronchial dysplasia in smokers [31].

Studies of other antioxidants, however, have been somewhat more promising. Selenium is a component of the antioxidant enzymes glutathione peroxidase and thioredoxin reductase, and therefore is thought to improve cellular defense against oxidative stress. *In vitro* studies indicated that selenium may cause regression of malignancy, and epidemiologic studies have suggested that increased selenium intake in populations with low average selenium levels may decrease the risk of lung cancer [32]. Conversely high levels of selenium may actually increase lung cancer risk [33].

When selenium was investigated for the prevention of skin cancer in a randomized, double-blind, placebo-controlled trial that included over 1,300 patients, the investigators found that the subjects who received 200 mcg selenium supplementation for 4.5 years had a 44% decrease in lung cancer incidence [34]. It seemed that the people with the lowest baseline selenium levels had the most significant decreased incidence. However, the SELECT (selenium and vitamin E cancer prevention trial) study, a randomized double-blind placebo-controlled, multi-center study of selenium for prostate cancer prevention in over 35,000 men, that included lung cancer incidence as a secondary endpoint, found that selenomethionine alone, or in combination with vitamin E, had no significant effect on lung cancer development [35,36,37]. Similarly, a chemoprevention trial in Linxian (China) of beta-carotene, alpha-tocopherol and selenium found no benefit to selenium supplementation [38], and a study of tertiary prevention with selenium in patients with previous lung-cancer resection showed no decrease in lung-cancer recurrence [28]. The variable results of these trials suggest that selenium may only be of benefit in those with low baseline levels, and that very high levels of selenium may even increase the risk of malignancy.

Cruciferous vegetables are natural sources antioxidants, and epidemiologic evidence has suggested that diets high in cruciferous vegetables (e.g., broccoli, cabbage, cauliflower, mustard greens, brussel sprouts, kale) may be associated with lower cancer incidence. A 2010 meta-analysis of the 30 studies looking at the association between cruciferous-vegetable consumption and lung-cancer risk found a weak inverse association [39]. In subsequent studies, researchers conducted two case-control analyses of the effects of cruciferous-vegetable intake on lung cancer risk. These later studies confirmed a decreased risk of lung cancer in those with the highest cruciferous vegetable intake, especially in current smokers [40,41].

Cruciferous vegetables are a major source of glucosinolates, which are precursors for isothiocyanates and indole-3-carbinol. These compounds exhibit several anti-carcinogenic properties. Indole-3-carbinol (I3C) modulates the PI3K/AKT/mTOR pathway (see below) and has been found to inhibit the development of carcinogen-induced adenocarcinoma in murine models [42]. Isothiocyanates may inhibit the bio-activation of tobacco carcinogens, such as polycyclic aromatic hydrocarbons and 4-(methylnitrosamino)-1-(3-pyridyl)-1-butanone [43]. They may also enhance excretion of carcinogenic metabolites before these metabolites can damage DNA [44,45]. Sulforaphane, a major isothiocyanate found in broccoli, has been found to induce cell-cycle arrest and apoptosis. Evidence suggests that individuals with variants in the glutathione-S-transferase gene, which plays an important role in xenobiotic metabolism and oxidative metabolism, may be particularly susceptible to the beneficial effects of increased isothiocyanate consumption. Murine studies showed that sulforaphane supplementation decreased benzo-(a)-pyrene induced lung damage and increased antioxidant levels and nuclear factor erythroid 2-related factor 2 (Nrf2) transcription [46,47]. These modulations by sulforaphane result in decreased carcinogen-induced stress. The results suggest an anti-initiating role of sulforaphane in experimentally induced lung cancer.

Dithiolethiones are organosulfur compounds that have antioxidant properties, and may be effective in cancer chemoprevention. In animal models, these compounds have been shown to have chemoprotective activity against the development of lung and other cancers [48,49,50,51]. Two of these compounds have been studied in human trials: Anethole dithiolethione (ADT; 5-[p-methoxyphenyl]-1,2-dithiole-3-thione) and oltipraz (5-[2-pyrazinyl]-4-methyl-1,2-dithiol-3-thione). Oltipraz was found to have too much toxicity for chemoprevention [52]. ADT is well tolerated, and in a randomized, double-blind, placebo-controlled, phase IIb trial was found to decrease both the development and progression of bronchial dysplasia in former smokers with known dysplasia [31]. ADT has multiple possible mechanisms of action, but the most prominent for chemoprevention appears to be its effects on glutathione. ADT stimulates glutathione synthesis via glutamyl cysteine synthetase, and increases glutathione-dependent enzyme activity, thus protecting against oxidative damage. ADT also displays free-radical scavenger properties that may protect cell membranes by inhibiting lipid peroxidation. It also modulates nuclear factor-kappa β, a redox-sensitive cytolytic transcription factor.

Green tea, made from the leaves of *Camellia sinensis*, or green tea extracts (GTE), are often proposed as cancer chemopreventives, primarily due to potent antioxidant properties. Observational evidence suggests an association between green-tea consumption and reduced lung cancer risk, although the data is conflicting [53]. Human trials have shown that high levels of green-tea consumption reduced oxidative damage in heavy smokers [54]. The active ingredients are thought to be green-tea polyphenols (GTPs) which, *in vitro*, can protect against DNA damage, promote apoptosis of tumor cells, and inhibit angiogenesis [55]. Recent studies suggest that GTE may also induce annexin-1 and inhibit COX-2 expression, suggesting that green tea may also have anti-inflammatory effects [56].

Mesna (2-mercaptoethane sulfonic acid) is an organosulfur compound used in combination with chemotherapy agents to decrease the toxicity of the agents’ metabolites. Mesna works as an anti-oxidant and detoxifies acrolein and other toxins by reaction of its sulfhydryl group with vinyl groups. It also increases urinary excretion of cysteine. Acrolein is one of the carcinogenic agents in cigarette smoke, suggesting that mesna may be useful as a chemopreventive agent in smokers, by acting as an antioxidant and detoxifying acrolein [57].

### 4.2. Anti-Inflammatories: Steroids, Cyclo-Oxygenase Inhibitors, Prostacyclin Analogs and PPARγ (Peroxisome Proliferator-Activated Receptor Gamma) Agonists, and HMG-CoA (Hydroxy-3-methyl-glutaryl Coenzyme A) Reductase Inhibitors (Statins)

Inflammation, especially prolonged inflammation, plays an important role in carcinogenesis. As mentioned, the respiratory tract is constantly exposed to a variety of environmental pathogens and toxins and has a potent immune response. This response includes the recruitment and activation of phagocytic and inflammatory immune cells, which produce oxidant mechanisms to fight infections or to promote the injury-repair process. However, the proliferative response to injury can also predispose to malignant transformation or progression. Multiple anti-inflammatory agents have been investigated for the chemoprevention of lung cancer.

Glucocorticoids have potent anti-inflammatory effects. They also alter genes involved in multiple signaling-pathways, particularly those involved in cell-cycle progression and MAP-kinase activation [58]. To investigate whether the use of inhaled steroids might decrease the risk of lung cancer, Parimon *et al*. looked at the records of over 10,000 veterans with COPD and found that those who had been treated with inhaled corticosteroids had a dose-dependent decreased risk of lung cancer [59]. Studies in murine carcinogenesis models have shown that glucocorticoids inhibit progression from hyperplasia to cancer, and can decrease tumor multiplicity and tumor load [60,61].

Prospective trials of inhaled glucocorticoids in high-risk smokers have been less convincing. In a randomized, placebo-controlled study of inhaled budesonide in smokers with persistent CT scan-detected lung nodules, there was a non-significant trend toward regression of non-solid and partially solid lesions after budesonide treatment [62]. Similarly, a randomized trial of inhaled budesonide in smokers with dysplasia of the bronchial epithelium did not induce regression [63]. 

Non-steroidal anti-inflammatory drugs (NSAIDs) act as inhibitors of the cyclooxygenase (COX) enzymes which convert arachidonic acid to prostaglandin E2. The COX enzymes play an important role in inflammation, and a large number of COX-2 dependent genes are involved in tumorigenesis [64,65]. COX-2 is often up-regulated in carcinoma-in-situ and non-small cell lung cancer [66]. In animal models, inhibition of cyclooxygenase activity decreases eicosanoid production and prevents the development of lung cancer [67]. As a result, many studies over the years have looked at NSAIDs as potential chemopreventive agents.

Aspirin is a nonselective inhibitor of both COX-1, a constitutive enzyme, and COX-2, an inducible enzyme, and may also cause cell-cycle arrest and apoptosis through alternate pathways. Since aspirin is widely used, several investigators have attempted to determine whether regular aspirin consumption decreases lung-cancer risk. In several different murine models, aspirin has been shown to prevent lung cancer, and this was associated with inhibition of COX-2, and induction of apoptosis [68,69,70,71,72].

The results of individual human studies have been variable [73,74,75]. However, a recent meta-analysis of 19 studies of NSAIDs and lung-cancer risk did suggest an overall benefit of taking aspirin (but not other NSAIDs) regularly for those at high risk of lung cancer [65]. Long-term follow up of trials of aspirin for prevention of cardiovascular disease also suggest a benefit for cancer prevention. Recent pooled analyses of a long-term post-trial follow up of cardiovascular disease and colorectal adenoma prevention studies demonstrated significant reductions in colorectal cancer incidence and mortality [76,77,78]. The 20-year risk of death due to all cancers, including lung, is consistently lower in the aspirin groups than in the control groups, and benefit increases with duration of treatment. The latent period before an effect on deaths was about five years for lung cancer, and was confined to adenocarcinoma. In fact, a recent report suggests that aspirin use may be associated with an increased risk of small-cell lung cancer [79]. In a prospective cohort study, NSAID use was associated with a small reduced risk of lung cancer, strongest for adenocarcinoma in men, and in long-term former smokers [80]. The only randomized, placebo-controlled trial of aspirin, the Women’s Health Study, found that women assigned low-dose aspirin (100 mg), taken every other day, had a borderline statistically-significant reduction in lung cancer risk [81].

Selective inhibitors of COX-2 have also been studied for cancer chemoprevention. Some of the agents investigated include celecoxib, etorcoxib and indomethacin. Recently, celecoxib given to current and former smokers was found to decrease Ki-67, a biomarker of proliferation, in bronchial epithelium and BAL cells after treatment [63,82]. Celecoxib has also been found to inhibit the production of PGE2 and reduce cancer incidence in high-risk patients. A recent study of another selective COX-2 inhibitor, etoricoxib, focused on the anti-angiogenic effect as another mechanism of chemoprevention [83]. However, an exhaustive review of the published literature on NSAIDs and cancer did not find any evidence for benefit or for risk of lung cancer with NSAID use [84].

Other NSAIDs that have recently been studied in relation to lung cancer risk include glucosamine/chondroitin [85] and tetramethylpyrazine, a COX2 inhibitor and component of traditional Chinese medicine [86].

Downstream in the cyclooxygenase pathway, the prostacyclin analogs also have anti-inflammatory effects, and have been investigated as chemopreventive agents. Prostacyclin analogs selectively increase peroxisome proliferator-activated receptor gamma (PPARγ) activity in epithelial cells and in non-small cell lung cancer (NSCLC) [87]. In NSCLC cell lines, activation of PPARγ inhibits cell growth [88]. In murine models, PPARγ over-expression prevents lung cancer. Iloprost, a long-lasting oral prostacyclin analog that inhibits lung tumorigenesis in carcinogen-exposed wild-type mice, was studied in a national trial and was found to decrease endobronchial dysplasia in former smokers [89].

Thiazolidinediones are oral PPARγ agonists used in diabetes management. They also induce 15-hydroxyprostaglandin dehydrogenase (15-PGDH), the enzyme that inactivates the anti-apoptotic and immunosuppressive PGE2 by conversion to 15 keto prostaglandins. Pioglitazone, a synthetic PPARγ ligand used in diabetes, induces tumor-cell apoptosis and has been found to prevent lung cancer in mouse models [90,91]. In one study of over 87,000 veterans treated for diabetes at ten Veterans Affairs medical centers, patients on thiazolidinediones had 33% lower incidence of lung cancer than those on other medications for diabetes management [92]. A meta-analysis completed this past year confirms a small, but significant decreased risk of lung (and other) cancers in diabetics treated with thiazolidinediones [93]. A review of patients with diabetes at the Cleveland Clinic found that those who had not been treated with metformin (see mTOR inhibitors section below) and/or thiazolidinediones were more likely to develop lung cancer [94]. An ongoing trial at the Denver VA is evaluating the effect of pioglitazone on lung cancer incidence in smokers [95].

The HMG-CoA reductase inhibitors are drugs that are widely prescribed for lipid disorders because of their ability to inhibit cholesterol synthesis. However, these drugs also affect multiple other pathways, including those involved in carcinogenesis, angiogenesis and immunomodulation [96,97]. Studies indicate that these drugs inhibit RhoA, suppress AKT activation and up-regulate TIMP-1, and therefore are potent anti-inflammatory agents and may also modulate DNA repair. Because of the possibility that these agents may inhibit carcinogenesis through their effects on multiple pathways, there is much interest in investigating their utility as chemopreventive agents. Epidemiologic studies looking at incidence of lung cancer in relation to statin use have had mixed results, although some studies—particularly in patients with COPD—seem to show a decreased risk of lung cancer [98,99]. Most recently, however, Jacobs *et al*. found no change in the risk of lung cancer after long-term statin use [100]. Murine studies did find that statins combined with green-tea polyphenols or glucocorticoids inhibited lung tumorigenesis as well as tumor-cell migration and metastasis [96].

### 4.3. PI3K/AKT/mTOR Pathway Inhibitors

Phosphatidylinositol 3-kinases (PI 3-kinases or PI3Ks) are a family of intracellular signal transducer enzymes that phosphorylate the hydroxyl group at the 3 position of the inositol ring of phosphatidylinositol and are involved in multiple cellular functions, including cell growth, proliferation, differentiation, motility, survival and intracellular trafficking. Many of these functions relate to the ability of class I PI 3-kinases to activate protein kinase B (PKB or AKT) in the PI3K/AKT/mTOR pathway. Tobacco smoke activates this pathway, and the pathway has been demonstrated to be activated in bronchial airways of smokers with airway dysplasia or lung cancer, and may precede the development of lung cancer [101]. The PI3K pathway can also be constitutively activated in tumors as a result of EGFR mutations, suppression of the tumor suppressor PTEN, PI3K gene mutations or increased PI3K gene copy. Mammalian target of rapamycin (mTOR) is a serine/threonine kinase that mediates the AKT signaling pathway.

Agents that affect the PI3K/AKT/mTOR pathway have potential as chemotherapeutic agents. Inhibitors of mTOR (rapamycin, sirolimus and metformin) induce cell-cycle arrest, and phase I studies of these agents for chemoprevention are planned [102,103,104]. However, the harmful side effects of some of these drugs will likely limit their utility as chemopreventive agents. One well-tolerated agent that inhibits mTOR is metformin, widely used for diabetes management. Metformin’s primary action is to inhibit hepatic glucose production and improve peripheral insulin-sensitivity. Evidence shows that metformin also inhibits PI3K/AKT/mTOR signaling. As mentioned above, observational studies have found a decreased incidence of lung cancer in diabetics treated with metformin compared with other agents [105,106], although not all studies confirm this observation [107,108].

Myo-inositol (*cis*-1,2,3,5-*trans*-4,6-cyclohexanehexol) is another well-tolerated inhibitor of the PI3K pathway. Inositol is an essential human cell nutrient and a component of cell membranes. The primary natural source of myo-inositol is inositol hexaphosphate, which is hydrolyzed by phytase in the gastrointestinal tract to free myo-inositol. Inositol hexaphospate is present in many foods, particularly whole grains and fruits. Myo-inositol, a glucose isomer, is a precursor in the phosphatidyl-inositol cycle, and the source of second messengers that regulate members of the protein kinase C family, intracellular calcium levels, and signal transduction. Derivatives of myo-inositol act as inhibitors of the PI3K pathway. *In vitro* studies have confirmed that myo-inositol specifically inhibits this pathway in lung-cancer cell lines. In addition, the activity of AKT [109] and PI3K [110] has been evaluated in the patients who had received myo-inositol, and regression of dysplasia correlates with decreased PI3K activity. In several studies, oral inositol inhibited lung tumorigensis in mice exposed to carcinogens [111]. It has been effective when given before, during and even immediately after exposure. Combining inositol with budesonide, dexamethasone, *N*-acetylcysteine and/or indole-3 carbinol increased efficacy even further [112]. In a phase I study of inositol in 26 smokers with bronchial dysplasia on autofluorescence bronchoscopy, Lam and colleagues found a significant increase in regression of dysplasia in patients treated with myo-inositol [113]. Ongoing studies continue to evaluate this agent for chemoprevention.

## 5. Conclusions

Many other strategies and agents have been investigated for lung cancer chemoprevention, though most have not progressed to the human clinical trial stage. Some of these strategies include STAT3 pathway inhibition (e.g., curcumin), cell-cycle arrest (e.g., gambogenic acid), hTERT silencing, miRNA modulation, iNOS suppression, chitin inhibition, angiotensin receptor blockade, TGF-β antagonism, VEGFR-2/EGFR inhibition (e.g., vandetanib), and Nrf2 activation. No agent has yet been proven sufficiently effective in human trials, but despite the disappointments in the field of lung cancer chemoprevention, there is reason to be optimistic. Many agents can inhibit lung carcinogenesis in animal models, and the development of intermediate markers will make the pursuit of human trials more feasible. As we learn more about altered pathways in pre-malignant lesions, more targeted or even personalized chemoprevention regimens may be devised. Improved lung cancer risk profiles will further define who should be selected for chemoprevention. Current models are based on general criteria of age, sex, tobacco smoke and asbestos exposure history [114], pulmonary function, family history, and presence of emphysema on CT [115,116]. Genetic testing will soon add additional criteria to define high-risk groups. It is likely that successful chemoprevention will require a combination of agents to target multiple pathways to carcinogenesis—both to minimize toxicity and maximize efficacy. Although avoidance of the major carcinogens is the surest way to decrease lung cancer incidence, chemoprevention for those at highest risk may eventually be possible. 
